# Ocular Surface Fluid: More than a Matrix

**DOI:** 10.3390/toxics12070513

**Published:** 2024-07-16

**Authors:** Ivan Šoša

**Affiliations:** Department of Anatomy, Faculty of Medicine, University of Rijeka, 51000 Rijeka, Croatia; ivan.sosa@uniri.hr

**Keywords:** conjunctiva, conjunctival sac, cornea, drugs of abuse, matrix, nasolacrimal duct, ocular surface fluid, tear

## Abstract

Although the eye can be subjected to therapeutic manipulation, some of its structures are highly inaccessible. Thus, conventional therapeutic administration pathways, such as topical or systemic routes, usually show significant limitations in the form of low ocular penetration or the appearance of side effects linked to physiology, among others. The critical feature of many xenobiotics is the drug gradient from the concentrated tear reservoir to the relatively barren corneal and conjunctival epithelia, which forces a passive route of absorption. The same is true in the opposite direction, towards the ocular surface (OS). With the premise that tears can be regarded as equivalent to or a substitute for plasma, researchers may determine drug concentrations in the OS fluid. Within this framework, a survey of scholarly sources on the topic was conducted. It provided an overview of current knowledge, allowing the identification of relevant theories, methods, and gaps in the existing research that can be employed in subsequent research. OS fluid (tears particularly) has enormous potential as a source of biological material for external drug screening and as a biomarker of various systemic diseases. Given the numerous alternate matrices, knowledge of their properties is very important in selecting the most appropriate specimens in toxicological analyses.

## 1. Introduction

Contemporary forensic toxicology uses a large number of matrices—components of a representative sample other than the analyte of interest. While blood and urine are commonly collected toxicological matrices and the oral route still prevails as the most popular route for systemic drug administration, there is a rising need for information helpful in the management of drug exposure [1,2,3]. A small amount of specimen found in the conjunctival sac is the paragon of the 3R principle (reduction, refinement, and replacement) [4].

A layer of the fluids on the ocular surface (OS) is basically three-dimensional; the OS sac is a space filled with a muco-aqueous pool (MAP) encircled in an ever-present lipid sealant lining the palpebral conjunctiva [5]. The rate of tear secretion is controlled by parasympathetic and sympathetic innervation [6]. In the above concept of the lipid film and MAP, tears are an essential component of the precorneal tear film. The conjunctival sac in humans measures approximately 30 µL. The mean tear volume is 6.5 ± 0.3 µL, and đ dropper bottles with ophthalmic solutions deliver drops of, on average, 40 µL [7,8]. Excessive fluid volume from the conjunctival sac must overflow onto the face if it is not sucked into the nasolacrimal duct—a membranous canal of about 18 mm in length [9]. Tear secretion in basal conditions occurs at a rate of about 1.2 µL/min (2 × 10^−8^ L/s). Tears enter the puncta at half of that rate, about 90% are reabsorbed through the nasolacrimal duct mucosa and 10% drain into the floor of the nasal cavity [10]. Blinking and tear flow can reduce this further so that less than five percent of conventional eye drops finally reach the systemic circulation [11,12]. Even prior to reaching systemic circulation, there are a number of anatomical barriers for a drug’s molecules to overcome [13]. 

## 2. Conjunctival Sac Fluid as a Biological Sample

**Figure 1:**
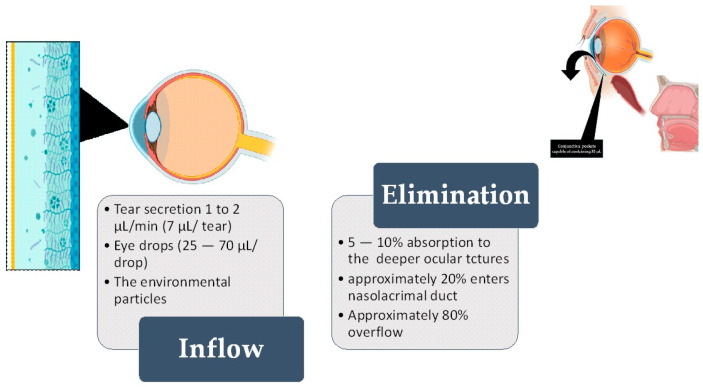
The left-hand side shows the inflow of fluid, while the right-hand side shows the elimination of fluid from the ocular surface (OS). The standard volume of the conjunctival “pocket” (fornix) is 30 µL [7,8]. Out of this, 7 µL is the constant tear film (or secreted tear) [20]. After the fluid passes through the conjunctiva and cornea (either transcellularly or paracellularly) through diffusion, 5–10% of the fluid is absorbed into eye structures and may enter the systemic circulation. As a single eye drop can have a volume ranging from 25 to 70 µL, it exceeds the remaining 23 µL of accessible conjunctival “pockets”. Most of the excess fluid overflows the eyelid, but around 10% is absorbed into the nasolacrimal duct [21].

Literature reports describe conjunctival sac fluid as a biological fluid extremely rich in some analytes secreted by the body [14,15]. Over the last few years, tear fluid analysis has garnered substantial attention in the field of diagnostics and sample collection methodology. Tears encompass a reservoir of biomarkers that assist in diagnosing many conditions [16]. Aside from that, conjunctival sac fluid is constantly exposed to the environment and its hazardous chemicals [17,18,19] (Figure 1).

### 2.1. Sampling

The transparent, three-layered fluid covering the surface of the eye (“tear film”), referred to as the OS fluid, is under-researched, though it is a promising alternative to traditional fluids [20,22]. In the work of Zhou et al., 2012 [19], tear samples were collected after making sure the subjects were wearing contact lenses. The sampling was performed using a unique standard Schirmer strip—a conventional sampling tool. These strips need to be frozen immediately after collection and until analysis. However, earlier research techniques, such as sponges, have also been used. Some researchers also use capillary tubes (though plastic rather than glass to minimize the risk of injury) for tear sampling, but this endeavor is time-consuming and requires expertise [23]. Schirmer strips are precisely diced and fully immersed in an elution buffer comprising 100 mM ammonium bicarbonate and protease inhibitor. Barmada and Shippy describe collecting the OS fluid via capillary action on phenol red thread. In their experiment, a color change length indicated the volume of fluid collected [22].

Currently, recommended devices (capillary tubes and Schirmer strips) are used to collect the OS without stimulation at a volume that allows for further laboratory analysis depending on the needs of the specific case and at the convenience of the person interpreting the analysis. In the cohort of Bachhuber et al., both of these methods were safe and well tolerated [23] (Table 1). Yao et al. recommend Schirmer strips even in microsampling for mass spectrometry analysis of human tears for the purpose of identifying drugs of abuse [24].

Even though blood is the most commonly used of all body fluids, tears are less complex and more accessible [38]. The volume of individual samples is restricted to 10–15 µL from each eye at a time, in any case.

### 2.2. Analysis

The analysis to date has been performed according to numerous protocols, potentially influencing the composition and quality of obtained samples. This inconsistency introduces considerable variability among studies.

Sensitive and accurate mass spectrometry (MS) techniques are the method of choice for this analysis of the OS fluid as the scarcity of samples can be further reduced in aged, dry eyes or in a long postmortem interval [39,40]. Better positioning in the pool of available techniques has positioned the MS methods among the most prominent new possible techniques for the analysis of specific molecules [41]. For instance, Yao et al. recommend Schirmer paper on noninvasive microsampling for direct mass spectrometry analysis of human tears and report on the analysis of cocaine and ketamine [24].

After thawing the ice to 4 °C before metabolite extraction, an aliquot of the tear fluid needs to be transferred into tubes for mass spectrometry and centrifuged. The yielded supernatants have to be pipetted out immediately and lyophilized. Finally, vortexed samples should undergo chromatographic separation. Further analytical processes vary significantly depending on the specific analytical requirements and the capabilities of the laboratory [16,42].

### 2.3. Method Validation

At present, both quick and easy sampling methods are equally preferred. Pieczyński et al. recommend both the use of Schirmer strips and microcapillary tubes as the cheapest and easiest methods for sampling OS fluid [43]. In the same vein, Tham et al. advise using either method for protein extraction [44].

The methods are evaluated through validation of their specificity, limit of detection (LOD), repeatability, accuracy, and matrix effect. The matrix can have a considerable effect on the way the analysis is conducted and the quality of the results obtained; such effects are called matrix effects. To this end, ocular surface fluid samples spiked with the target analytes at varying concentrations have to be employed.

In performing analyses needed for calculations of those parameters, the mixture of artificial tears and sodium hyaluronate eye drops is advised as a blank control, at least in some works [42]. Some authors claim that the physical properties of artificial tears significantly differ from those of ocular surface fluid, and this should be taken into account to understand a potential caveat [45].

## 3. Drugs in Ocular Surface Fluid

Conjunctiva and nasal mucosa are the chief routes for the instilled dose to enter the systemic blood circulation [46].

Although the eye can be subjected to therapeutic manipulation, some of its structures are highly inaccessible. Thus, conventional therapeutic administration pathways, such as topical or systemic routes, usually show significant limitations in the form of low ocular penetration or the appearance of side effects linked to physiology, among others.

With the premise that tears can be regarded as equivalent to or a substitute for plasma, researchers may determine drug concentrations in the ocular surface fluid.

**Figure 2:**
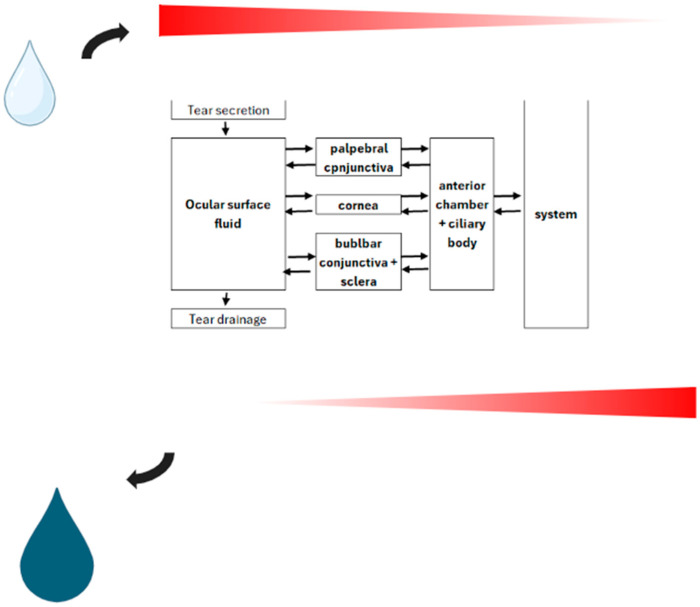
Schematic representation of topical drug delivery model with passive diffusion employed as indicated along the concentration gradient across a bidirectional barrier.

OS fluid represents another means of drug elimination (Figure 2). Some drugs can be excreted to varying extents in some unusual matrices. In many studies, the ratio of the tear (i.e., ocular surface fluid) concentration to plasma concentration (T/P ratio) has been challenged for many drugs. Although the drug’s concentration in serum reasonably correlates to that in ocular surface fluid, the correlation of the T/Pb ratio to the acid dissociation constant (pKa) value is less impressive. Van Haeringen (1986) [47] calculated r^2^ = 0.46 at *p* = 0.000 and a goodness-of-fit R^2^ = 0.21. pKa and pH are related in the Henderson–Hasselbalch equation. Most medications are not concentrated solutions, so the following approximation (Equation (1)) is valid:

(1) $$pH=pKa+log10\left(\frac{\left[A–\right]}{\left[HA\right]}\right)$$

meaning pH is equal to the sum of the pKa value and the log of the equilibrium concentrations of the conjugate acid–base pair ([A⁻] and [HA]) used to create the buffer solution

The pH is a measure of the acidity or alkalinity of a solution. It can affect a drug’s ability to cross biological membranes, and changes in this property can influence the degree of ionization and, consequently, a drug’s absorption and distribution. For that reason, theoretically predicted and expected ratios of drug concentration in plasma and ocular surface fluid do not always correspond to those found in clinical settings. In more acidic drugs, these ratios were lower than theoretically expected. This could be due to the lower lipid solubility of these drugs. The same was the case with tetracyclines and their probable entry into tears [48,49]. Unionized drugs and those with high pKa values have a clinically proven T/P ratio in good agreement with the theoretically expected distribution ratios. The discrepancies of higher values than expected might be explained by active transport mechanisms or by higher concentrations in arterial blood plasma during the absorption phase. This showcases the value of pKa in any drug’s distribution [50,51].

### 3.1. Ever-Present Lipid Sealant Film (Precorneal Compartment)

The lipid sealant film possesses optical characteristics like transparency and refracting capability, and its morphological features ensure that it is anchored to the eyeball. This film also refines vision [52]. Most of the modern literature describes tear fluids in the OS as a “film”, “the tear film”. The tear film itself is a cohesive, viscoelastic entity extending into the retro-palpebral pouches [53]. These are narrow pockets where palpebral and bulbar conjunctiva meet in the lower eyelid, with deeper recesses in the upper eyelid [54]. Drugs administered topically to this site are absorbed across the cornea, and the pre-corneal tear film determines the absorption based on the lipophilicity of the drug. The lipid layer in this pre-corneal tear film reduces surface tension. Beneath that, the corneal stroma is a highly hydrophilic layer that behaves like a highly viscous fluid (with a viscosity of about 1.5-times that of water) [55]. The corneal stroma is rate-limiting for the transport of lipophilic drugs [56]. The lipid sealant has been confirmed to be an actual “thin film”, much thinner than the associated MAP. This sealant resides between the OS epithelium and the surface lipid film of the eyelids. The base of the MAP is anchored to the varied epithelia of its OS sac, creating a dacron/surface complex (DSC) rather than a simple interface. Even the lipids of the stratum corneum from the eyelids are important regulators of the skin permeability of topically applied drugs [57,58]. This is the standard route of topically administered ocular medication.

Aside from that, drugs administered topically may be eliminated from this “pocket” through mechanisms like the initial outflow of the drug by blinking and the nasolacrimal route [59,60].

### 3.2. Routes in the Interface between the Functioning Eye and Our Environment

A number of limitations, both physiological and anatomical, allow the absorption of only a tiny portion of topically administered xenobiotics. Factors such as drainage, lacrimation, tear rheology, and corneal and conjunctival permeability play an essential role in poor ocular bioavailability of dosage forms. The cornea and conjunctiva are the most significant tissue barriers that limit ocular drug absorption after its application on the surface of the eye. For this reason, it is essential to understand drug permeability at the cornea and conjunctiva and its impact on ocular drug absorption.

The relatively impermeable corneal barrier and rapid drainage of the instilled solution protecting the eye are distinct mechanisms used in regard to systemic absorption and topical penetration of a drug. The cornea is the favored route for topical ocular drug absorption due to its anatomical structure. Even though the corneal epithelium is relatively impermeable, it is more permeable than the stratum corneum of the skin [57]. At the same time, it acts as a bidirectional barrier. As a protective barrier, it protects from the invasion of foreign substances and acts as a barrier to ion transport [61]. Due to the relatively exposed structure, drugs with a molecular size of up to 500,000 Da (with molecular dimensions up to about 20 nm) can usually diffuse beneath that epithelium [62]. Drugs penetrate through the corneal epithelium either by the transcellular (lipophilic drugs) or paracellular (hydrophilic drugs) route [63]. Ocular medications applied topically undergo passive diffusion along a concentration gradient via a transcellular or paracellular route [64].

Considering the physicochemical features of the ocular barriers, drugs diffuse across the barrier from a low concentration to high concentration based on Fick’s first law of diffusion (Equation (2)) [65].

(2) $$\frac{dCd}{dt}=kdcA\left(Cd-Cy\right)$$

where

did/dt = the net drug moving from tears to the cornea per unit of time;

A = the area of the tear–corneal interface;

Cd = concentration of the drug in tears;

Cy = concentration of the drug in the cornea;

Kdc = the permeability constant from tears to the cornea.

The paracellular route involves passive or altered diffusion through intercellular spaces, and it is the primary permeation mechanism across the cornea. However, there are instances of corneal transport that involve the Na’-K’-ATPase pump and a stereospecific carrier-mediated transport system (such as l-lysine).

On the other hand, the conjunctiva of the eyelids (palpebral conjunctiva) is continuous with the mucus membrane of the globe (bulbar conjunctiva). Together, they form a thin, vascularized space for nonproductive conjunctival drug absorption. This surface area of 18 cm^2^ is considered to be nonproductive only from the standpoint of topical ocular medication [66]. However, the presence of conjunctival vasculature can cause significant drug loss in the systemic circulation, thereby lowering ocular bioavailability [67]. The conjunctival sac has a surface area 17-times larger than the cornea, and its epithelium has intercellular tight junctions leakier than the cornea [62,67]. Be it as it may, conjunctival tight junctions of the superficial epithelium are the main barrier to the penetration of xenobiotics across conjunctiva [68].

For the most part, drug absorption from the conjunctival sac leads to systemic drug absorption. For instance, 70–80% of the timolol dose is absorbed systemically, and this causes systemic adverse drug effects [69]. Even though many ocular drugs hit the iris or ciliary body, trans-conjunctival permeation is used as a method of delivery.

Ex vivo studies of animal corneal and conjunctival drug permeability, in addition to the c models for drug permeability, were employed to predict the impact of ocular barriers on ocular and systemic drug absorption from the fluid of the OS [46].

### 3.3. Nasal Mucosa

The OS fluid is drained from the OS through the nasolacrimal duct—a part of the nasolacrimal system connecting the OS with the nasal punctum located on the floor of the nasal vestibule [70]. Nasolacrimal duct obstruction has been described in chronic intranasal cocaine abuse [71], and this most certainly infringes on the ocular drug deployed by the nasal cavity.

When OS fluid is used as a matrix and as a route of administration, the active pharmaceutical ingredient (drug or xenobiotic) has to reach the absorbing surface. In the eye, this is the conjunctiva. In the nasal cavity, the active pharmaceutical ingredient needs to be deposited on the epithelial membrane and absorbed before disappearing or being degraded [12,72,73].

- The first surface or barrier for active pharmaceutical ingredient absorption is the mucus layer of the nasal cavity. The molecules of active pharmaceutical ingredients are dissolved here; otherwise, they trespass through the mucus layer before being swept away by mucociliary activity or succumbing to enzymatic degradation [74];
- A double-layered, stratified columnar epithelium resting on a broad basement membrane lies beneath the luminal vascular plexuses. In the apical part, epithelial cells contain large lipid droplets and secretory vacuoles. Microvilli face epithelial cells, and some tufts of kinociliae are also visible. The plexus is embedded in the helical system of different connective tissue fibers, which is comparable to a cavernous body [75,76]. The superior part of this helical system is the 12 mm intraosseous portion, and its lower 5 mm part is the membranous portion;
- The last surface that an active pharmaceutical ingredient encounters before its absorption into the blood is the capillary endothelium. This step is essential for systemically targeting active pharmaceutical ingredients [73].

In brief, the active pharmaceutical ingredient reaches the nasal cavity, where absorption can also occur through the nasal mucosa. Up to 80% of the applied xenobiotic(s) may diffuse into the systemic circulation by crossing the highly vascularized nasopharyngeal mucosa. The xenobiotic does not undergo any first-pass metabolism, as the blood drainage from this region is not associated with the portal circulation [65,77]. Notwithstanding, the route of absorption used by any active pharmaceutical ingredient depends on its physicochemical properties.

## 4. The “New” Matrix

Aside from the tears, which are a filtrate of the blood plasma (though tear fluid is contributed by the lachrymal gland, as well), OS fluid consists of any material resulting from the passive diffusion, such as ocular medications instilled into the pocket between the conjunctiva palpebralis and conjunctiva bulbaris and any substance from the environment. Blood plasma, a fundamental component in tears, circulates the entire body, reaching all organs and tissues, and it therefore provides a wealth of relevant clinical information on seemingly unrelated body parts [78,79]. For example, a selective beta blocker, practolol, was associated with oculomucocutaneous syndrome long ago [80]. In some cases, limited oral bioavailability of some compounds and the considerable difficulty of the intravenous approach (fear of needles, infections, etc.) have prompted the search for more effective routes for the systemic delivery of xenobiotics [11]. For instance, most general anesthetics decrease the tear flow and are associated with corneal ulcers and epithelial disruption [81]. In contrast, ketamine anesthesia produces profuse lacrimation, which may be of help [82]. Rifampin is lipo-soluble, so it passes into tears well [83].

These are examples that amply demonstrate how systemic drugs may induce external changes to the eye via adverse effects on tear flow and tear constituents. Examples of creative illicit use include the recreational misuse of eye drop tropicamide and the use of tetrahydrozoline-containing eye drops to alter positive urine drug tests [84,85,86]. In cases like this, OS fluid is more than a matrix; it is a route of entrance. In regard to other drugs of abuse, noninvasive monitoring of drugs of abuse is currently focused on saliva testing [87]. Interestingly, cocaine could not be detected in the tears of humans exposed to aerosolized cocaine [88].

Unlike blood, the analysis of tears requires a sample of minimal size with minimal invasiveness in collection. Subsequent analysis is rapid, and technology is constantly undergoing advancements. Dualde et al. report the effectiveness of an untargeted method for the identification of unknown substances using an ultra-high-performance liquid chromatography–high-resolution mass spectrometry (UHPLC-HRMS) system [89]. They developed an intelligent data acquisition approach (AcquireX DS-dd-MS2) coupled to an automated data processing software (Compound Discoverer™ 3.2) as well. Another advantage of OS fluid in the biological context of this matrix is the much lower concentration of some molecules in OS fluids compared with blood.

The use of OS fluid analysis as part of routine postmortem healthcare should enhance its integration into the clinical casework and improve the essential development and advancement of analytical technologies.

## 5. Conclusions

OS fluid (tears particularly) has enormous potential as a source of biological material for external drug screening and as a biomarker of various systemic diseases.

Given the numerous alternate matrices, knowledge of their properties is very important in selecting the most appropriate specimens in toxicological analyses. OS fluid provides additional information and advantages in comparison to blood and urine testing and can be collected and analyzed when blood and urine are not available.

Some critical analytical and biochemical challenges prohibit the comprehensive implementation of tear analysis in routine post-mortem healthcare. Methods of preanalytical, analytical, and postanalytical handling of OS fluid are needed in order to obtain more significant amounts of information from OS fluid samples.

Further exploiting the potential of OS fluid analysis should be a goal on the medical research radar. Issues like sample collection, the detection window, and the complexity of sample preparation/analysis are yet to be addressed.

## Figures and Tables

**Table 1:** Model sampling and analysis methods used for identifying and/or quantifying various analytes in the ocular surface (OS) fluid.

Analysis Method	Exposure	Collection Method	Chemical or Contaminant	Reference
Agar diffusion assay	Air pollution	Filter paper	Lysozymes	Berra et al., 2015 [25]; Galperín et al., 2018 [26]
ELISA	Tobacco smoke	Capillary tube	Lysine adducts	Rummenie et al., 2008 [27]
ELISA	Tobacco smoke	Capillary tube	Cytokines	Rummenie et al., 2008 [27]
Ethanol assay kit	Alcohol	Capillary tube	Ethanol	Kim et al., 2012 [28]
GC-MS	Air pollution	Schirmer paper	Lipids	Gutierrez et al., 2019 [29]
ICP-MS	Trace elements	Capillary tube	Trace elements	Chen et al., 2022 [30]
Immunoassay	Mold	Capillary tube	Complement components	Peltonen et al., 2008 [31]
Immunoassay	Air pollution	Capillary tube	Cytokines	Matsuda et al., 2015 [32]; Jing et al., 2022 [33]
LC-MS	Ozone	Capillary tube	Lipids	Paananen et al., 2015 [34]
PIXE	Air pollution	Schirmer paper	Trace elements	Girshevitz et al., 2022 [35]
PSMS	Smoke	Schirmer paper	Nicotine	Yao et al., 2020 [24]
PSMS	Aerosols	Schirmer paper	Salbutamol	Yao et al., 2020 [24]
PSMS	Drugs of abuse	Schirmer paper	Cocaine, ketamine	Yao et al., 2020 [24]
PSMS	Volatile organic compounds	Schirmer paper	Arginine, glucose	Yao et al., 2020 [24]
SEM/EDS	Particulate matter	Schirmer paper	Particulate matter	Avula et al., 2017 [36]
SEM/EDS	Indoor environment	Schirmer paper	Particulate matter	Kaplan et al., 2019 [37]

## Data Availability

All relevant data are within the manuscript.

## References

[1] Kunsman G.W., Hartman R.L., Levine B.S., Kerrigan S. (2020). Human Performance Toxicology. Principles of Forensic Toxicology.

[2] Levine B.S., Levine B.S., Kerrigan S. (2020). Postmortem Forensic Toxicology. Principles of Forensic Toxicology.

[3] de Campos E.G., da Costa B.R.B., Dos Santos F.S., Monedeiro F., Alves M.N.R., Santos Junior W.J.R., De Martinis B.S. (2022). Alternative matrices in forensic toxicology: A critical review. Forensic Toxicol..

[4] Louzao M.C., Costas C. (2024). 4.1. 1 The Three Rs. Environmental Toxicology: Non-Bacterial Toxins.

[5] Cher I. (2012). Fluids of the ocular surface: Concepts, functions and physics. Clin. Exp. Ophthalmol..

[6] Masoudi S. (2022). Biochemistry of human tear film: A review. Exp. Eye Res..

[7] Costa A.X.d., Gama R.M.d., Kitadai S.P.S., Andrade E.P.d., Ferro G.B.R., Gomes J.Á.P. (2015). Drop volume of artificial tear solutions: Pharmacoeconomic study. Rev. Bras. Oftalmol..

[8] Pflugfelder S.C., Gumus K., Feuerman J., Alex A. (2017). Tear Volume-based Diagnostic Classification for Tear Dysfunction. Int. Ophthalmol. Clin..

[9] Schaudig U., Shams P., Ducasse A. (2024). Anatomy and Physiology of the Lacrimal System. Oculoplastic, Lacrimal and Orbital Surgery: The ESOPRS Textbook: Volume 1.

[10] Price K.M., Richard M.J. (2009). The tearing patient: Diagnosis and management. Am. Acad. Ophthalmol..

[11] Ahmed S., Amin M.M., Sayed S. (2023). Ocular Drug Delivery: A Comprehensive Review. AAPS PharmSciTech.

[12] Tai J., Han M., Lee D., Park I.H., Lee S.H., Kim T.H. (2022). Different Methods and Formulations of Drugs and Vaccines for Nasal Administration. Pharmaceutics.

[13] Le T., Aguilar B., Mangal J.L., Acharya A.P. (2022). Oral drug delivery for immunoengineering. Bioeng. Transl. Med..

[14] Caselli E., Soffritti I., Lamberti G., D’Accolti M., Franco F., Demaria D., Contoli M., Passaro A., Contini C., Perri P. (2020). Anti-SARS-CoV-2 IgA Response in Tears of COVID-19 Patients. Biology.

[15] Secchiero P., Lamberti G., Corallini F., Melloni E., Guarnotta C., Sebastiani A., Zauli G. (2009). Conjunctival sac fluid contains elevated levels of soluble TRAIL: Implications for the anti-tumoral surveillance of the anterior surface of the eye. J. Cell. Physiol..

[16] Thomas K.M., Ajithaprasad S., Mithun N., Pavithran M.S., Chidangil S., Lukose J. (2024). Raman spectroscopy assisted tear analysis: A label free, optical approach for noninvasive disease diagnostics. Exp. Eye Res..

[17] Suzuki T., Sutani T., Nakai H., Shirahige K., Kinoshita S. (2019). Human microbiome of eyelid skin, conjunctival sac, and meibum of the meibomian gland. Investig. Ophthalmol. Vis. Sci..

[18] Amini P., Okeme J. (2023). Tear Fluid as a Matrix for Biomonitoring Environmental and Chemical Exposures. Curr. Environ. Health Rep..

[19] Zhou L., Zhao S.Z., Koh S.K., Chen L., Vaz C., Tanavde V., Li X.R., Beuerman R.W. (2012). In-depth analysis of the human tear proteome. J. Proteom..

[20] Chang A.Y., Purt B. (2021). Biochemistry, Tear Film.

[21] Van Santvliet L., Ludwig A. (2004). Determinants of eye drop size. Surv. Ophthalmol..

[22] Barmada A., Shippy S.A. (2020). Tear analysis as the next routine body fluid test. Eye.

[23] Bachhuber F., Huss A., Senel M., Tumani H. (2021). Diagnostic biomarkers in tear fluid: From sampling to preanalytical processing. Sci. Rep..

[24] Yao Y.N., Di D., Yuan Z.C., Wu L., Hu B. (2020). Schirmer Paper Noninvasive Microsampling for Direct Mass Spectrometry Analysis of Human Tears. Anal. Chem..

[25] Milevoj Kopcinovic L., Culej J., Jokic A., Bozovic M., Kocijan I. (2020). Laboratory testing of extravascular body fluids: National recommendations on behalf of the Croatian Society of Medical Biochemistry and Laboratory Medicine. Part I—Serous fluids. Biochem. Med..

[26] Nioi M., Napoli P.E., Demontis R., Locci E., Fossarello M., d’Aloja E. (2021). Postmortem Ocular Findings in the Optical Coherence Tomography Era: A Proof of Concept Study Based on Six Forensic Cases. Diagnostics.

[27] Vianney Ramírez Ojeda S., Hernandez Mier C., Peter C., Kamil Hakan D. (2024). Postmortem Interval Ocular Indicators. Contemporary Issues in Clinical Bioethics—Medical, Ethical and Legal Perspectives.

[28] Urban P.L. (2016). Quantitative mass spectrometry: An overview. Philos. Trans. A Math. Phys. Eng. Sci..

[29] Huang Z. (2024). Trace analysis of steroid hormones in tear films via liquid chromatography-high resolution mass spectrometry. Anal. Methods.

[30] Berra M., Galperin G., Dawidowski L., Tau J., Marquez I., Berra A. (2015). Impact of wildfire smoke in Buenos Aires, Argentina, on ocular surface. Arq. Bras. Oftalmol..

[31] Galperin G., Berra M., Marquez M.I., Mandaradoni M., Tau J., Berra A. (2018). Impact of environmental pollution on the ocular surface of Sjogren’s syndrome patients. Arq. Bras. Oftalmol..

[32] Rummenie V.T., Matsumoto Y., Dogru M., Wang Y., Hu Y., Ward S.K., Igarashi A., Wakamatsu T., Ibrahim O., Goto E. (2008). Tear cytokine and ocular surface alterations following brief passive cigarette smoke exposure. Cytokine.

[33] Kim J.H., Kim J.H., Nam W.H., Yi K., Choi D.G., Hyon J.Y., Wee W.R., Shin Y.J. (2012). Oral alcohol administration disturbs tear film and ocular surface. Ophthalmology.

[34] Gutierrez M.L.A., Colman Lerner J.E., Giuliani D.S., Porta A.A., Andrinolo D. (2019). Comparative study of tear lipid composition in two human populations with different exposure to particulate matter in La Plata, Argentina. Environ. Sci. Pollut. Res. Int..

[35] Chen Y.J., Chen Y.Y., Lai C.H. (2022). Clinical association between trace elements of tear and dry eye metrics. Sci. Rep..

[36] Peltonen S., Kari O., Jarva H., Mussalo-Rauhamaa H., Haahtela T., Meri S. (2008). Complement activation in tear fluid during occupational mold challenge. Ocul. Immunol. Inflamm..

[37] Matsuda M., Bonatti R., Marquezini M.V., Garcia M.L., Santos U.P., Braga A.L., Alves M.R., Saldiva P.H., Monteiro M.L. (2015). Lacrimal Cytokines Assessment in Subjects Exposed to Different Levels of Ambient Air Pollution in a Large Metropolitan Area. PLoS ONE.

[38] Jing D., Jiang X., Zhou P., Ren X., Su J., Hao R., Zhang M., Wan Y., Li X. (2022). Evidence of air pollution-related ocular signs and altered inflammatory cytokine profile of the ocular surface in Beijing. Sci. Rep..

[39] Paananen R.O., Rantamaki A.H., Parshintsev J., Holopainen J.M. (2015). The Effect of Ambient Ozone on Unsaturated Tear Film Wax Esters. Investig. Ophthalmol. Vis. Sci..

[40] Girshevitz O., Cohen-Sinai N., Zahavi A., Vardizer Y., Fixler D., Goldenberg-Cohen N. (2022). Trace Elements in Tears: Comparison of Rural and Urban Populations Using Particle Induced X-ray Emission. J. Pers. Med..

[41] Avula A., Galor A., Blackwelder P., Carballosa-Gautam M., Hackam A.S., Jeng B., Kumar N. (2017). Application of Scanning Electron Microscopy with Energy-Dispersive X-Ray Spectroscopy for Analyzing Ocular Surface Particles on Schirmer Strips. Cornea.

[42] Kaplan C., Galor A., Blackwelder P., Hackam A.S., Jeng B.H., Menendez D., Kim S.J., Kumar N. (2019). Human Ocular Surface Particulate Composition in the Clinical Versus Home Environment. Cornea.

[43] Pieczynski J., Szulc U., Harazna J., Szulc A., Kiewisz J. (2021). Tear fluid collection methods: Review of current techniques. Eur. J. Ophthalmol..

[44] Tham M.L., Mahmud A., Abdullah M., Md Saleh R., Mohammad Razali A., Cheah Y.K., Mohd Taib N., Ho K.L., Mahmud M., Mohd Isa M. (2023). Tear Samples for Protein Extraction: Comparative Analysis of Schirmer’s Test Strip and Microcapillary Tube Methods. Cureus.

[45] Recchioni A., Mocciardini E., Ponzini E., Tavazzi S. (2022). Viscoelastic properties of the human tear film. Exp. Eye Res..

[46] del Amo E.M. (2022). Topical ophthalmic administration: Can a drug instilled onto the ocular surface exert an effect at the back of the eye?. Front. Drug Deliv..

[47] van Haeringen N.J. (1985). Secretion of drugs in tears. Curr. Eye Res..

[48] Agwuh K.N., MacGowan A. (2006). Pharmacokinetics and pharmacodynamics of the tetracyclines including glycylcyclines. J. Antimicrob. Chemother..

[49] Sigmund A.B., Ward D.A., Cox S.K., Hendrix D.V.H. (2020). Tear film concentrations of topically applied 0.5% oxytetracycline ointment in normal canine eyes. Vet. Ophthalmol..

[50] Abuhelwa A.Y., Williams D.B., Upton R.N., Foster D.J. (2017). Food, gastrointestinal pH, and models of oral drug absorption. Eur. J. Pharm. Biopharm..

[51] Gaohua L., Miao X., Dou L. (2021). Crosstalk of physiological pH and chemical pKa under the umbrella of physiologically based pharmacokinetic modeling of drug absorption, distribution, metabolism, excretion, and toxicity. Expert. Opin. Drug Metab. Toxicol..

[52] Montes-Mico R., Cervino A., Ferrer-Blasco T., Garcia-Lazaro S., Madrid-Costa D. (2010). The tear film and the optical quality of the eye. Ocul. Surf..

[53] Semiz F., Lokaj A.S., Tanriverdi G., Caliskan G., Hima-Musa N., Semiz C.E. (2022). Fresh Human Myopic Lenticule Intrastromal Implantation for Keratoconus Using SMILE Surgery in a Long-term Follow-up Study: Ultrastructural Analysis by Transmission Electron Microscopy. J. Refract. Surg..

[54] Shumway C.L., Motlagh M., Wade M. (2024). Anatomy, Head and Neck, Eye Conjunctiva. StatPearls.

[55] Santana C.P., Matter B.A., Patil M.A., Silva-Cunha A., Kompella U.B. (2023). Corneal Permeability and Uptake of Twenty-Five Drugs: Species Comparison and Quantitative Structure-Permeability Relationships. Pharmaceutics.

[56] Lanier O.L., Manfre M.G., Bailey C., Liu Z., Sparks Z., Kulkarni S., Chauhan A. (2021). Review of Approaches for Increasing Ophthalmic Bioavailability for Eye Drop Formulations. AAPS PharmSciTech.

[57] See G.L.L. (2020). Unlocking the Potential of Eyelid Skin-Based Drug Delivery: A Filipino Pharmaceutical Scientist’s Journey. J. Pharm. Sci. Technol. Jpn..

[58] Derakhshani A., Hasani S., Navaei T. (2023). Hemocompatible polymers for medical applications. Handbook of Polymers in Medicine.

[59] Fayyaz A., Ranta V.P., Toropainen E., Vellonen K.S., Valtari A., Puranen J., Ruponen M., Gardner I., Urtti A., Jamei M. (2020). Topical ocular pharmacokinetics and bioavailability for a cocktail of atenolol, timolol and betaxolol in rabbits. Eur. J. Pharm. Sci..

[60] Agrahari V., Mandal A., Agrahari V., Trinh H.M., Joseph M., Ray A., Hadji H., Mitra R., Pal D., Mitra A.K. (2016). A comprehensive insight on ocular pharmacokinetics. Drug Deliv. Transl. Res..

[61] Sridhar M.S. (2018). Anatomy of cornea and ocular surface. Indian J. Ophthalmol..

[62] Mofidfar M., Abdi B., Ahadian S., Mostafavi E., Desai T.A., Abbasi F., Sun Y., Manche E.E., Ta C.N., Flowers C.W. (2021). Drug delivery to the anterior segment of the eye: A review of current and future treatment strategies. Int. J. Pharm..

[63] Khalil A., Barras A., Boukherroub R., Tseng C.-L., Devos D., Burnouf T., Neuhaus W., Szunerits S. (2024). Enhancing paracellular and transcellular permeability using nanotechnological approaches for the treatment of brain and retinal diseases. Nanoscale Horiz..

[64] Tashima T. (2024). Ocular Drug Delivery into the Eyes Using Drug-Releasing Soft Contact Lens. Future Pharmacol..

[65] Bartlett J.D., Jaanus S.D. (2007). Clinical Ocular Pharmacology.

[66] Zhang X., Zhang Y., Luo G., Xie R., Li H., Cheng W. (2021). Discussion on the Development of New Ophthalmic Dosage Forms of Ganciclovir and Its Pre-Development Evaluation. CONVERTER.

[67] Ramsay E., Del Amo E.M., Toropainen E., Tengvall-Unadike U., Ranta V.P., Urtti A., Ruponen M. (2018). Corneal and conjunctival drug permeability: Systematic comparison and pharmacokinetic impact in the eye. Eur. J. Pharm. Sci..

[68] Agarwal R., Iezhitsa I., Agarwal P., Abdul Nasir N.A., Razali N., Alyautdin R., Ismail N.M. (2016). Liposomes in topical ophthalmic drug delivery: An update. Drug Deliv..

[69] Cimolai N. (2019). Neuropsychiatric Adverse Events from Topical Ophthalmic Timolol. Clin. Med. Res..

[70] Lassaline M., Reed S.M., Bayly W.M., Sellon D.C. (2018). Disorders of the Eye and Vision. Equine Internal Medicine.

[71] Nitro L., Pipolo C., Fadda G.L., Allevi F., Borgione M., Cavallo G., Felisati G., Saibene A.M. (2022). Distribution of cocaine-induced midline destructive lesions: Systematic review and classification. Eur. Arch. Otorhinolaryngol..

[72] (2018). Methods in Toxicology. Toxicology and Risk Assessment.

[73] Kumar V., Bansal V., Madhavan A., Kumar M., Sindhu R., Awasthi M.K., Binod P., Saran S. (2022). Active pharmaceutical ingredient (API) chemicals: A critical review of current biotechnological approaches. Bioengineered.

[74] Leal J., Smyth H.D.C., Ghosh D. (2017). Physicochemical properties of mucus and their impact on transmucosal drug delivery. Int. J. Pharm..

[75] Yartsev V.D., Atkova E.L., Ekaterinchev M.A. (2023). Topographic and anatomical features of the nasolacrimal duct obstruction due to radioiodine treatment. Int. Ophthalmol..

[76] Munjal M., Munjal S., Vohra H., Prabhakar A., Kumar A., Singh S., Soni A., Gupta S., Agarwal M. (2021). Anatomy of the lacrimal apparatus from a rhinologist’s perspective: A review. Int. J. Otorhinolaryngol. Head Neck Surg..

[77] Law S.K., Lee D.A. (2020). Ocular pharmacology. Glaucoma Medical Therapy-Principles and Management.

[78] Morton S., Crucian B., Hagan S., Satyamitra M., Daily A. Pilot study on the investigation of tear fluid biomarkers as an indicator of ocular, neurological, and immunological health in astronauts. Proceedings of the NASA Human Research Program Investigators’ Workshop (HRP IWS 2018).

[79] Tomeckova V., Tkacikova S., Talian I., Fabriciova G., Hovan A., Kondrakhova D., Zakutanska K., Skirkova M., Komanicky V., Tomasovicova N. (2023). Experimental Analysis of Tear Fluid and Its Processing for the Diagnosis of Multiple Sclerosis. Sensors.

[80] Mann R.D. (2007). An instructive example of a long-latency adverse drug reaction—Sclerosing peritonitis due to practolol. Pharmacoepidemiol. Drug Saf..

[81] Ioannides J., Parker J., Kumaratunga V., Preston J., Donaldson D., MacFarlane P., Hartley C. (2022). A prospective, masked, randomized, controlled superiority study comparing the incidence of corneal injury following general anesthesia in dogs with two methods of corneal protection. Vet. Ophthalmol..

[82] Rascón-Martínez D.M., Carrillo-Torres O., Ramos-Nataren R.G., Rendón-Jaramillo L. (2018). Advantages of ketamine as a perioperative analgesic. Rev. Médica Del Hosp. Gen. De México.

[83] Suresh A.B., Rosani A., Patel P., Wadhwa R. (2023). Rifampin. StatPearls [Internet].

[84] Hasson H. Generalized Onset Tonic-Clonic Seizures. MedLink. https://www.medlink.com/articles/generalized-onset-tonic-clonic-seizures.

[85] Bersani F.S., Corazza O., Simonato P., Mylokosta A., Levari E., Lovaste R., Schifano F. (2013). Drops of madness? Recreational misuse of tropicamide collyrium; early warning alerts from Russia and Italy. Gen. Hosp. Psychiatry.

[86] Bellman V., Ukolova A., Erovichenkova E., Lam S., Srivastava H.K., Bruce J., Burgess D.M. (2022). Abuse of tropicamide eye drops: Review of clinical data. Braz. J. Psychiatry.

[87] Vindenes V., Lund H.M., Andresen W., Gjerde H., Ikdahl S.E., Christophersen A.S., Oiestad E.L. (2012). Detection of drugs of abuse in simultaneously collected oral fluid, urine and blood from Norwegian drug drivers. Forensic Sci. Int..

[88] Roque Bravo R., Faria A.C., Brito-da-Costa A.M., Carmo H., Mladenka P., Dias da Silva D., Remiao F., On Behalf of The Oemonom R. (2022). Cocaine: An Updated Overview on Chemistry, Detection, Biokinetics, and Pharmacotoxicological Aspects including Abuse Pattern. Toxins.

[89] Dualde P., Miralles P., Peris-Martinez C., Yusa V., Coscolla C. (2023). Untargeted analysis and tentative identification of unknown substances in human tears by ultra-high performance liquid chromatography-high resolution mass spectrometry: Pilot study. J. Chromatogr. B Analyt. Technol. Biomed. Life Sci..

